# Gut to Brain Dysbiosis: Mechanisms Linking Western Diet Consumption, the Microbiome, and Cognitive Impairment

**DOI:** 10.3389/fnbeh.2017.00009

**Published:** 2017-01-30

**Authors:** Emily E. Noble, Ted M. Hsu, Scott E. Kanoski

**Affiliations:** ^1^Human and Evolutionary Biology Section, Department of Biological Sciences, University of Southern CaliforniaLos Angeles, CA, USA; ^2^Neuroscience Program, University of Southern CaliforniaLos Angeles, CA, USA

**Keywords:** neuroinflammation, insulin, gut bacteria, sugar, fat, endotoxin, hippocampus

## Abstract

Consumption of a Western Diet (WD) that is high in saturated fat and added sugars negatively impacts cognitive function, particularly mnemonic processes that rely on the integrity of the hippocampus. Emerging evidence suggests that the gut microbiome influences cognitive function via the gut-brain axis, and that WD factors significantly alter the proportions of commensal bacteria in the gastrointestinal tract. Here we review mechanisms through which consuming a WD negatively impacts neurocognitive function, with a particular focus on recent evidence linking the gut microbiome with dietary- and metabolic-associated hippocampal impairment. We highlight evidence linking gut bacteria to altered intestinal permeability and blood brain barrier integrity, thus making the brain more vulnerable to the influx of deleterious substances from the circulation. WD consumption also increases production of endotoxin by commensal bacteria, which may promote neuroinflammation and cognitive dysfunction. Recent findings also show that diet-induced alterations in gut microbiota impair peripheral insulin sensitivity, which is associated with hippocampal neuronal derrangements and associated mnemonic deficits. In some cases treatment with specific probiotics or prebiotics can prevent or reverse some of the deleterious impact of WD consumption on neuropsychological outcomes, indicating that targeting the microbiome may be a successful strategy for combating dietary- and metabolic-associated cognitive impairment.

## Introduction

Substantial evidence has linked consumption of a Western Diet (WD), defined here as diets consisting of both high levels of fat (35–60% total kcal) and added sugars, with cognitive dysfunction (Molteni et al., 2002, 2004; Kanoski et al., 2007, 2010; Kanoski and Davidson, 2011; Davidson et al., 2012; Francis and Stevenson, 2013; Baym et al., 2014; Beilharz et al., 2014, 2016a,b; Noble et al., 2014; Hsu et al., 2015; Khan et al., 2015b; Noble and Kanoski, 2016). The hippocampus, a brain region associated with the control of certain learning and memory processes, is particularly vulnerable to the deleterious effects of WD intake (Kanoski and Davidson, 2011; Baym et al., 2014; Davidson et al., 2014). The mechanisms through which WD consumption impacts the brain are not completely understood, however emerging research has implicated the gut-brain axis as playing a critical role. The gut microbiome (the collective genome of microbes residing in the gastrointestinal tract) has a substantial impact on brain function (Bercik et al., 2011; Davari et al., 2013; Hsiao et al., 2013; Bruce-Keller et al., 2015). Moreover, the gut microbiome is profoundly affected by dietary factors (de La Serre et al., 2010; David et al., 2014; Noble et al., 2017). In this review we raise the hypothesis that the microbiome is a critical link between WD consumption and neurocognitive dysfunction. Putative mechanisms connecting WD consumption, microbiota alterations, and cognitive impairment include barrier integrity (gastrointestinal tract, neurovascular), neuroinflammation, and impaired insulin signaling. Though each topic is considered individually, it is likely that these and other biological outcomes associated with WD consumption work in concert to impact brain function.

## Western diet, hippocampal function, and gut microbiota

Extensive evidence from rodent (reviewed in Kanoski and Davidson, 2011) and human studies (Kalmijn et al., 2004; Francis and Stevenson, 2011; Baym et al., 2014) reveals that consumption of a WD is linked with impaired hippocampal-dependent learning and memory function. Due to the obesity-promoting nature of WD, it is difficult to discern the relative contribution of dietary factors and obesity on cognitive outcomes. However, while obesity *per-se* is associated with reduced hippocampal volume (Jagust et al., 2005) and impaired hippocampal function (Li et al., 2002; Winocur et al., 2005; Khan et al., 2015a), evidence shows that a WD negatively impacts hippocampal function independent of obesity. For example, hippocampal-dependent spatial memory impairments have been reported after only 3 (Kanoski and Davidson, 2010) or 9 (Murray et al., 2009) days of consuming a WD, despite similar body weights between animals compared to standard chow-fed controls. Similar to WDs, high fructose diets can also impair hippocampal-dependent learning and memory in rodents independent of obesity (Hsu et al., 2015; Agrawal et al., 2016; Meng et al., 2016; Noble and Kanoski, 2016). Together these data suggest that dietary factors common in a WD have the capacity to impart cognitive dysfunction after only a brief exposure, and independent of severe metabolic impairments.

While much progress has been made in elucidating the neurobiological mechanisms underlying WD-associated cognitive impairment (reviewed in Kanoski and Davidson, 2011; Beilharz et al., 2015), few reports consider the gut microbiome, which consists of an estimated 100 trillion microorganisms that reside in the host GI tract (Bäckhed et al., 2005). The gut microbiome has emerged as a major contributor to cognitive health (Gareau et al., 2011; Bajaj et al., 2012; Bruce-Keller et al., 2015; Desbonnet et al., 2015; Fröhlich et al., 2016) and is affected by dietary factors (Daniel et al., 2014; David et al., 2014; Bruce-Keller et al., 2015; Magnusson et al., 2015; Noble et al., 2017). For example, consumption of a WD reduces populations in the phylum *Bacteroidetes* and increases *Firmicutes* and *Proteobacteria* (Hildebrandt et al., 2009; Zhang et al., 2012) in adult rodents. Importantly, these shifts have been associated with cognitive impairments. Magnusson et al., observed that both high fat (45% kcal from fat) and high sugar (70% kcal from carbohydrate) diets elevated levels of *Clostridiales* (Phylum: *Firmicutes*) and reduced levels of *Bacteroidales* (Phylum: *Bacteroidetes*) in rodents, changes that correlated to poor cognitive flexibility (Magnusson et al., 2015). Recent evidence supports a functional link between the gut microbiome and WD-induced cognitive dysfunction. Bruce-Keller and colleagues revealed that fecal/cecal transplantation from adult mice fed a WD to antibiotic pre-treated mice fed a control diet increased anxiety and stereotypic activity and impaired contextual fear conditioning (Bruce-Keller et al., 2015). While the bacterial species or combination of species responsible for the behavioral effects was not identifiable due to the whole microbiome transfer approach, the bacteria *Akkermansia muciniphila* were substantially (5.4-fold) reduced by the WD, whereas *Bilophila* sp. were elevated in the WD group and barely detectable in the control group (Bruce-Keller et al., 2015). Notably, *A. muciniphila* promotes insulin sensitivity and reduces metabolic endotoxemia in mice (Shin et al., 2014) and is negatively associated with metabolic disease, intestinal inflammatory diseases, and autism in humans (reviewed in Derrien et al., 2016). Conversely, *Bilophila* are positively associated with inflammatory intestinal diseases (Devkota et al., 2012; Jia et al., 2012) and may contribute to neurocognitive abnormalities by promoting inflammation, though this has not been directly tested.

Recent studies examined the individual contributions of particular macronutrients from a WD on the gut microbiome. For example, a high-fat, carbohydrate-free diet (72% kcal from fat) reduces *Bifidobacteria* (Cani et al., 2007, 2008), which have been shown modulate intestinal barrier function and reduce endotoxin levels in the gut (Griffiths et al., 2004; Wang et al., 2006). Conversely, Jena and colleagues found that a low-fat chow diet supplemented with a 65% w/v fructose solution had no effect on levels of *Bifidobacteria* (Jena et al., 2014). However, using more moderate concentrations that model commonly consumed sugar sweetened beverages, recent data from our group reveal that *Bifidobacteria* were elevated following free access to the sugar solutions (and chow and water) relative to controls that were not given sugar (Noble et al., 2017), suggesting that sugar-induced gut microbiome alterations are dependent on the carbohydrate concentration. Data from our recent study further show that consuming the sugar solutions altered gut bacteria at every phylogenetic level, including significant group effects in ~25% of gut bacteria at the family level. Similar to animals on very high doses of fructose solution (Jena et al., 2014), our data revealed that sugar consumption elevated *Enterobacteriaceae*, which are associated with gut (Lupp et al., 2007) and brain inflammation, and poor cognition in hepatic encephalopathy (Bajaj et al., 2012; Ahluwalia et al., 2016). Surprisingly, rodents consuming 11% concentrations of sugar solutions have elevated levels of *Lactobacilli* (Noble et al., 2017), which are anti-inflammatory, whereas rodents consuming much higher concentrations of sugar solutions (Jena et al., 2014) or dietary fat (Lecomte et al., 2015) have reduced levels of *Lactobacilli*. Notably, *Lactobacilli* facilitate short chain fatty acid transport (Kumar et al., 2015). Short chain fatty acids (SCFAs) alter human health and their reduced absorption may be one of the mechanisms by which diet impacts cognitive health via the gut microbiome, a concept reviewed below.

## Short chain fatty acids

SCFAs such as acetate, propionate, and butyrate are produced in the gut by microbial-mediated fermentation of indigestible carbohydrates, such as resistant starch and non-starch polysaccharides from cereals, vegetables, and fruits (MacFarlane and MacFarlane, 2011). The production of SCFAs is significantly reduced in humans within days following a dietary change from a complex carbohydrate rich plant-based diet to an animal based diet high in saturated fat and low in complex carbohydrates (David et al., 2014). Similarly, rodents fed a WD had reduced levels of SCFAs, including acetic, propionic, isobutyric, and isovaleric acids (Ojo et al., 2016) as well as butyric and valeric acids (Berger et al., 2014), compared with controls fed a low-fat chow diet. When taken in context with previously discussed studies demonstrating that short-term consumption of a WD significantly impacts cognitive function (Kanoski and Davidson, 2010; Beilharz et al., 2014, 2016a,b), the rapid alteration of SCFAs as a putative contributor to WD-induced cognitive dysfunction is temporally feasible.

While the majority of the SCFAs in portal circulation are metabolized by the liver, SCFAs produced in the distal colon bypass portal circulation and reach the brain through circulation (reviewed in MacFabe, 2012). In the brain, SCFAs have a neuroprotective effects (Sun et al., 2015), for example, the salt of the SCFA butyric acid, sodium butyrate, promotes cell proliferation and differentiation in the dentate gyrus, increases the expression of brain-derived neurotrophic factor (BDNF) and glia-derived neurotrophic factor (GDNF), and improves memory performance in the novel object recognition task (Wu et al., 2008; Stefanko et al., 2009; Intlekofer et al., 2013; Yoo et al., 2015). These neuroprotective effects of sodium butyrate potentially occur through the inhibition of histone deacetylase (HDAC), which is known to prevent the transcription of BDNF and GDNF (Wu et al., 2008). Butyrate also has anti-inflammatory actions in the gut and brain by preventing the induction of the inflammatory cytokine TNFα by the endotoxin lipopolysaccharide (LPS) via the suppression of nuclear factor κB (Segain et al., 2000). Taken together, SCFAs produced by gut bacteria (whose levels are reduced by WD consumption; Berger et al., 2014) may affect brain health directly via HDAC inhibition in the brain, or indirectly by reducing systemic inflammation in the gut. Butyrate has also been shown to stabilize hypoxia-inducible factor (HIF; Kelly et al., 2015), which is critical for maintaining gut barrier integrity and protecting against the influx of potentially harmful toxins, a topic discussed in more depth below.

## Gut and neurovascular barrier integrity

Emerging research is revealing that gut microbiota have potent effects on gut permeability (Cani et al., 2007, 2008, 2009; Lam et al., 2012; Pendyala et al., 2012; Tulstrup et al., 2015; Maffeis et al., 2016; Mokkala et al., 2016; Müller et al., 2016) and blood-brain barrier integrity (Braniste et al., 2014), both of which are negatively impacted by WD intake and proposed mechanisms underlying WD induced cognitive impairments (Kanoski et al., 2010; Davidson et al., 2012; Hsu and Kanoski, 2014; Ouyang et al., 2014; Hargrave et al., 2016; Stranahan et al., 2016). Several studies discussed here support a causal relationship between WD-mediated gut microbiota alterations, the gut/neurovascular barrier integrity, and hippocampal function.

The gut barrier consists of a specialized, semi-permeable mucosal, and epithelial cell layers that are reinforced by tight junction proteins. Among other functions, this barrier serves to regulate nutrient and water entry and prevents the entry of harmful compounds into extra-luminal tissues (for review see Turner, 2009). WD consumption impairs gut permeability, which in turn allows for the influx of adverse substances and may ultimately contribute to the development of metabolic disorders, and cognitive dysfunction. For example, in humans there is a strong association between obesity, gut permeability, and systemic inflammation (Maffeis et al., 2016; Rainone et al., 2016). In rodents, WD intake decreases levels of the tight junction protein ZO-1 and transepithelial resistance in the proximal colon, both markers of gut barrier dysfunction (Lam et al., 2012). A compromised gut barrier makes the intestinal tract potentially vulnerable to the gram-negative bacteria-derived LPS, which upon excess entry into circulation promotes endotoxemia and systemic inflammation (Griffiths et al., 2004; Cani et al., 2007, 2008; Tsukumo et al., 2007). Indeed, mice maintained on a WD for 4 weeks exhibit a ~three-fold increase in circulating LPS levels with concurrent increased intestinal permeability, as reflected by reduced mRNA expression of tight junction proteins ZO-1 and occludin, as well as elevated plasma levels of a gavaged fluorescent molecule (FITC-dextran) that is typically unable to cross the gut barrier (Cani et al., 2008). This study further demonstrated that antibiotic treatment attenuated obesity-induced endotoxemia, thus providing potential physiological links between WD, the gut microbiome, and gut barrier integrity (Cani et al., 2008).

The blood-brain barrier (BBB) consists of a structural complex of endothelial cells, pericytes, and glial cells that encompass microvasculature networks within the central nervous system. It serves as a critical regulator for the entry of blood-derived nutrients and compounds required for healthy brain function, while simultaneously precluding the entry of potentially harmful blood-derived toxins. Importantly, WD intake is associated with BBB damage, which may be causally related to WD-induced cognitive dysfunction (Kanoski et al., 2010; Freeman et al., 2011; Davidson et al., 2012; Freeman and Granholm, 2012; Pallebage-Gamarallage et al., 2012; Hargrave et al., 2016; Stranahan et al., 2016). For example, in a study from Kanoski et al. (2010), rats maintained on a WD for 90 days exhibited a leaky BBB in the hippocampus and reduced mRNA expression of the tight junction proteins claudin-5 and claudin-12. These negative BBB outcomes were accompanied by impairments in hippocampal-dependent memory tasks, suggesting that WD-induced BBB damage may be causally related to cognitive deficits. Davidson and colleagues extended this work (Davidson et al., 2012) by showing that rats prone to obesity are more susceptible to WD-induced BBB damage and cognitive impairment compared to obesity resistant animals. Moreover, the magnitude of BBB damage and memory impairment depends on both obesity susceptibility and the duration of WD exposure (Hargrave et al., 2016). Collectively, these data provide strong evidence linking WD intake, BBB integrity, and hippocampal dysfunction.

Braniste et al. (2014) illuminate an association between gut microbiome perturbation and impaired BBB integrity. Infrared-labeled immunoglobulin antibody (IgG2b; normally precluded from brain parenchyma) injected into pregnant germ-free (microbiome-free) mouse dams was abundant in the brains of their mouse embryos compared to the embryos of pathogen-free (microbiome-intact) dams, suggesting that maternal gut microbiome has strong influences on the offspring's BBB integrity. Moreover, compared to adult pathogen-free mice, adult germ-free mice exhibited impaired BBB integrity evidenced by increased brain uptake of tail-vein injected radio labeled ligand [11C] raclopride and increased presence of Evans blue dye (normally precluded from BBB penetration) in brain parenchyma following circulatory injections. Interestingly, transfer of pathogen-free fecal matter to germ-free mice attenuated BBB damage, reflected by the increased expression of tight junction proteins. These data implicate an important role for the gut microbiome in regulating BBB integrity. Whether gut microbiome perturbations associated with WD consumption are causally related to WD-associated barrier dysfunction and memory impairments require further investigation.

## Neuroinflammation

Rodent studies have consistently shown that chronic consumption of a WD elevates levels of neuroinflammatory markers, which are associated with impaired cognition (Pistell et al., 2010; Puig et al., 2012; Herculano et al., 2013; Camer et al., 2015; Hsu et al., 2015; Ledreux et al., 2016). In conjunction with cognitive impairments, rats fed a WD have increased neuroinflammation in both the hippocampus (Puig et al., 2012; Herculano et al., 2013; Hsu et al., 2015; Ledreux et al., 2016) and in the cortex (Pistell et al., 2010; Camer et al., 2015). Moreover, clinical reports implicate a positive association between circulating inflammatory factors and cognitive decline in humans (Sweat et al., 2008; Sellbom and Gunstad, 2012). A WD may impact neuroinflammation and cognitive outcomes in part via altering levels of gut bacteria, as certain gut bacteria stimulate the innate immune system to elevate inflammatory cytokines in the brain (for review see Sankowski et al., 2015).

One putative mechanism through which WD influences gut bacteria and imparts hippocampal dysfunction involves elevated levels of endotoxin and accompanying inflammatory cytokines. WD consumption elevates levels of inflammatory endotoxins such as LPS (Cani et al., 2007; Amar et al., 2008; Bruce-Keller et al., 2015), and elevated levels of microbiome-derived LPS in circulation stimulate inflammatory pathways. Additionally, gut microbiota directly stimulate the production of the proinflammatory cytokines IL-1β and TNFα (Heumann et al., 1994), which have been shown to impair hippocampal-dependent memories in rodents (Rachal Pugh et al., 2001; Goshen et al., 2007; Hein et al., 2010).

In addition to elevating levels of endotoxin producing bacteria, a WD may affect neuroinflammation by reducing levels of anti-inflammatory commensal gut bacteria. Bruce-Keller et al. (2015) demonstrated that WD fecal/cecal transplant recipient mice were normal weight, yet had elevated levels of endotoxin and neuroinflammatory markers accompanied by impaired cognitive function. The bacterial species *A. muciniphila* was reduced by the diet (Bruce-Keller et al., 2015); a species that is negatively associated with inflammation (Schneeberger et al., 2015). Similarly, anti-inflammatory *Lactobacilli* are reduced by WD factors (Jena et al., 2014; Lecomte et al., 2015) and supplementation with *Lactobacillus helveticus* prevents spatial memory impairment in WD-fed mice lacking the anti-inflammatory cytokine IL-10 (Ohland et al., 2013). One method by which members of the *Lactobacilli* family and other commensal bacteria may reduce systemic inflammation is by improving insulin sensitivity (Simon et al., 2015), a concept reviewed in the following section.

## Insulin

Insulin, produced in pancreatic beta cells and released in response to conditioned cephalic cues or circulating metabolites, crosses the BBB via a saturable transporter, and insulin receptors are present in neurons and primarily localized to synapses (Zhao and Alkon, 2001). Levels of insulin receptor are particularly concentrated in the hippocampus (Havrankova et al., 1978) where insulin signaling improves cognitive function and neuronal plasticity (Biessels et al., 1998; Kamal et al., 2000; Grillo et al., 2009, 2015; Biessels and Reagan, 2015). WD-induced peripheral insulin resistance is associated with impaired cognitive function and synaptic plasticity in rats (Elias et al., 1997; Grodstein et al., 2001; Hiltunen et al., 2001; Yaffe et al., 2004; Stranahan et al., 2008; Pavlik et al., 2013; Gao et al., 2015) and the risk for developing Alzheimer's disease or dementia in humans (Ott et al., 1999; Arvanitakis et al., 2004; Luchsinger et al., 2004; Cukierman et al., 2005; Rönnemaa et al., 2008).

Insulin impacts neurological health via multiple mechanisms. One of the functions of CNS insulin is to phosphorylate α-amino-3-hydroxy-5-methyl-4- isoxazolepropionic acid (AMPA) receptors, which leads to increased hippocampal long-term potentiation (LTP; Adzovic and Domenici, 2014). Another mechanism through which insulin may improve cognitive function is by reducing neuroinflammation. For example, intracerebroventricular injection of insulin attenuates LPS-induced elevations in IL-1β and improves spatial memory impairment in young rats (Adzovic et al., 2015). Insulin has a similar anti-inflammatory function in the periphery, where it has been shown to reduce the systemic inflammatory response to endotoxin (Jeschke et al., 2004). Thus, if gut microbiota impact cognitive function in part via the modulation of inflammatory responses, or by elevating levels of peripheral or central endotoxin, then insulin may provide protection against gut microbiome-mediated cognitive dysfunction in insulin sensitive individuals. Given that long-term exposure to *ad libitum* fructose (15% w/v in water) impairs insulin receptor function in the hippocampus and reduced hippocampus-dependent spatial memory (Agrawal et al., 2016), a harmful synergy may occur through which WD intake both increases neuroinflammation (as reviewed above) and impairs central insulin sensitivity, thereby preventing insulin from attenuating inflammatory responses and associated adverse neuronal outcomes.

Recent findings directly link gut microbiota and CNS/peripheral insulin sensitivity. In humans, an intra-duodenal microbiome transfer from lean healthy donors to individuals with impaired insulin sensitivity improves insulin sensitivity in the recipients (Vrieze et al., 2012). Interestingly, transferring the fecal microbiome from obese or lean discordant human twin pairs to mice resulted in impaired glucose metabolism in the mouse if the transfer came from an obese twin (Ridaura et al., 2013). In mice, antiobiotic-induced microbiome depletion improves peripheral insulin sensitivity caused by a WD (Suárez-Zamorano et al., 2015). The effect of gut microbiota on peripheral insulin sensitivity begins at the level of the intestinal mucosa, as WD-induced insulin resistance is prevented by blocking live intestinal bacteria from translocating into the blood and tissues where they generate an inflammatory response (Amar et al., 2011). Bacterial translocation preceded WD-induced insulin resistance, and required functioning Nod1 and CD14 receptors, which bind to gram-negative bacteria. Furthermore, the translocation of bacteria and the insulin resistance were preventable when animals were treated with the probiotic *Bifidobacterium Animalis*, which specifically prevents translocation of *Enterobacteriaceae* (Amar et al., 2011). Taken together, commensal bacteria may alter peripheral insulin sensitivity in a mechanism that likely involves inflammatory signaling and/or bacterial translocation from the gut into the periphery.

Whether consuming a WD promotes cognitive dysfunction through modifying gut microbiota that impair insulin receptor signaling remains to be determined. However, one study revealed that probiotic treatment normalized spatial memory deficits and improved hippocampal LTP in a streptozocin rat model of diabetes (Davari et al., 2013), suggesting that gut microbiota can improve cognitive dysfunction due to reduced insulin production. Interestingly, evidence suggests that commensal gut bacteria may enhance insulin sensitivity and cognitive function by modulating the production and/or secretion of the incretin hormones glucagon-like peptide—1 (GLP-1). SCFAs, such as butyrate, produced by commensal gut bacteria act on G protein coupled receptors to stimulate GLP-1 secretion (Tolhurst et al., 2012). Hwang and collegues showed that antibiotics reduced the proportion of *Bacteriodetes* and *Firmicutes* in mice, which resulted in attenuated pancreatic islet hypertrophy and improved insulin and glucose tolerance through a GLP-1 signaling pathway (Hwang et al., 2015). Importantly, GLP-1 signaling promotes hippocampal neural plasticity and improved memory function (During et al., 2003; McClean et al., 2010; Li et al., 2012). Taken together, these collective data suggest that commensal gut bacteria modulate insulin sensitivity via multiple mechanisms, which may be related to WD-induced hippocampal dysfunction.

## Concluding remarks

**Figure 1:**
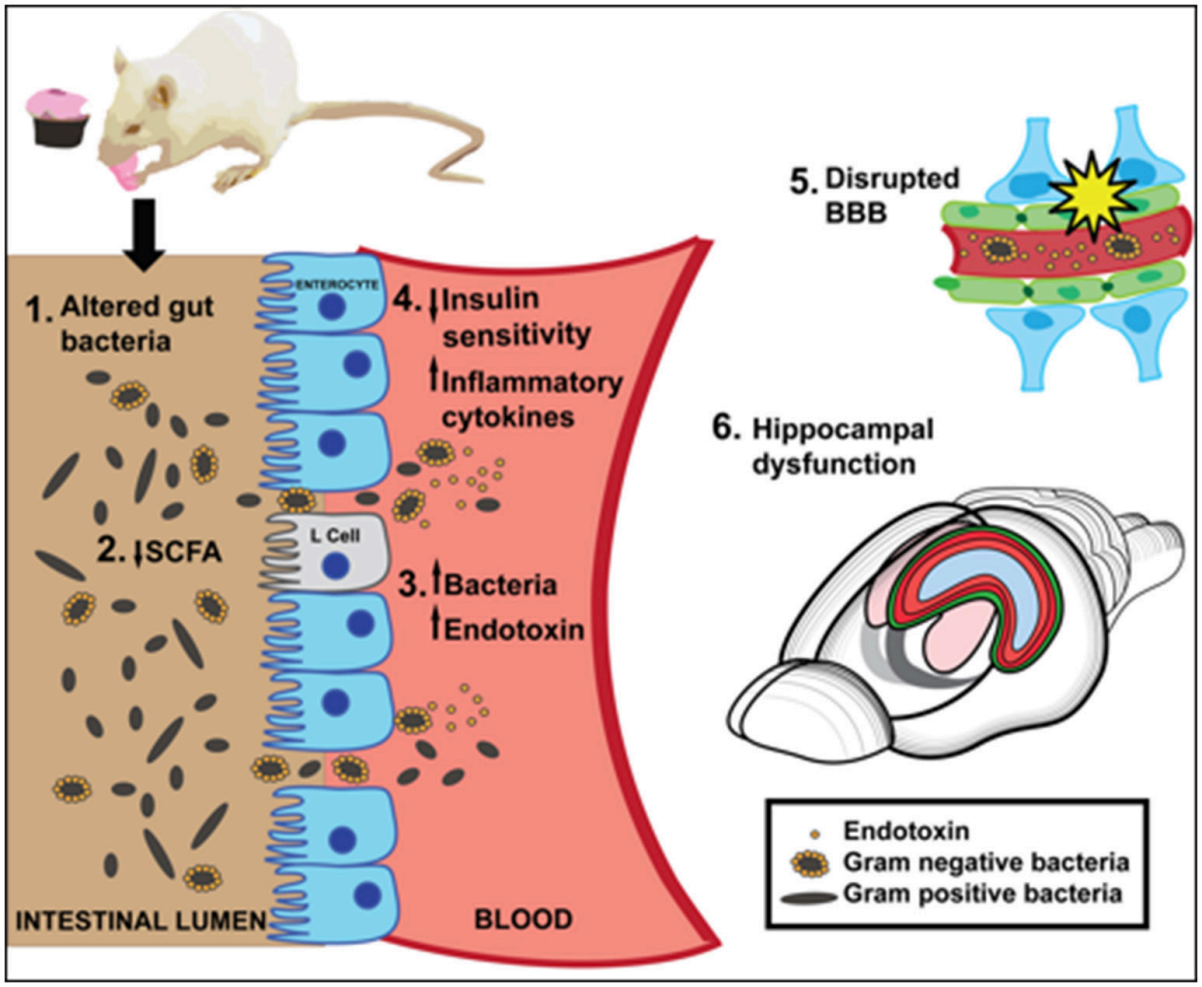
**A summary of putative mechanisms linking Western Diet (WD) consumption, the gut microbiome, and cognitive dysfunction**. [1] A high fat/high sugar WD diet alters gut bacteria [2] WD reduces short chain fatty acids (SCFA), which may impair neuroprotection or anti-inflammatory effects in the gut. SCFAs affect insulin signaling by stimulating L cell production of GLP-1. [3] WD may impair intestinal barrier and promote translocation of endotoxin-producing gram negative bacteria into the blood. [4] Inflammatory cytokines and/or reduced insulin sensitivity caused by WD-induced gut bacteria may negatively affect hippocampal function and memory. [5] A WD impairs BBB integrity, which may be caused in part by altered gut microbiota. [6] WD consumption significantly impairs hippocampal dependent learning and memory.

Several neurobiological mechanisms link WD consumption with gut microbiome alterations that potentially contribute to WD-mediated cognitive dysfunction, including reduced SCFA production, compromised barrier integrity, neuroinflammation, and peripheral and/or central insulin receptor resistance (Figure 1). Consuming a WD promotes endotoxemia, which is linked with memory impairment, either via translocation of gram-negative bacteria into circulation, and/or by impairing the permeability of the gut barrier. Both gut microbiota and WD intake have been shown to impair the permeability of the BBB, however mechanistic studies linking WD intake, BBB integrity, and the gut microbiome are required. WD-associated microbiota alterations impair peripheral insulin sensitivity, which is strongly linked with central insulin resistance and hippocampal dysfunction. In addition, insulin protects against peripheral inflammatory responses to endotoxin, and may prevent the deleterious effects imparted by WD-mediated bacterial production of endotoxins in insulin sensitive individuals.

Overall we present multiple pathways through which WD-induced microbiome alterations can impact neurocognitive function. Mechanistic studies examining these putative gut brain axis pathways may facilitate the development of therapies that target the microbiome (probiotics, prebiotics, antibiotics, or microbiota transfer) to treat neurobiological and cognitive dysfunction associated with WD intake and associated metabolic disorders.

## Author contributions

All three authors contributed to the idea for the manuscript. EN and TH wrote and edited the manuscript. SK edited the manuscript, provided vital input to shape the manuscript, and contributed to the writing. All three authors approved the final manuscript.

## Funding

This work was supported by grant number DK104897 from the National Institute of Diabetes and Digestive and Kidney Diseases and also by the University of Southern California Diabetes and Obesity Research Institute (Awarded to SK).

### Conflict of interest statement

The authors declare that the research was conducted in the absence of any commercial or financial relationships that could be construed as a potential conflict of interest.
